# Assessment of Anti-Inflammatory and Antimicrobial Potential of Ethanolic Extract of *Woodfordia fruticosa* Flowers: GC-MS Analysis

**DOI:** 10.3390/molecules26237193

**Published:** 2021-11-27

**Authors:** Agnieszka Najda, Aarti Bains, Prince Chawla, Anil Kumar, Sebastian Balant, Magdalena Walasek-Janusz, Dariusz Wach, Ravinder Kaushik

**Affiliations:** 1 Department of Vegetable and Heerbal Crops, University of Life Science in Lublin, 51A Doświadczalna Street, 20-280 Lublin, Poland; agnieszka.najda@up.lublin.pl (A.N.); sebastianbalant@o2.pl (S.B.); magdalena.walasek@up.lublin.pl (M.W.-J.); 2 Department of Biotechnology, CT Institute of Pharmaceutical Sciences, South Campus, Jalandhar 144020, Punjab, India; 3 Department of Food Technology and Nutrition, Lovely Professional University, Phagwara 144411, Punjab, India; 4 School of Bioengineering and Food Technology, Shoolini University, Solan 173229, Himachal Pradesh, India; kumaranil_16@rediffmail.com; 5 Subdepartment of Plant Nutrition, University of Life Science in Lublin, 28 Głęboka Street, 20-612 Lublin, Poland; dariusz.wach@up.lublin.pl; 6 School of Health Sciences, University of Petroleum and Energy Studies, Dehradun 248007, Uttrakhand, India; ravinder_foodtech2007@rediffmail.com

**Keywords:** antioxidant activity, chromatography, flower extract, antimicrobial activity

## Abstract

Currently, the potential utilization of natural plant-derived extracts for medicinal and therapeutic purposes has increased remarkably. The current study, therefore, aimed to assess the antimicrobial and anti-inflammatory activity of modified solvent evaporation-assisted ethanolic extract of *Woodfordia fruticosa* flowers. For viable use of the extract, qualitative analysis of phytochemicals and their identification was carried out by gas chromatography–mass spectroscopy. Analysis revealed that phenolic (65.62 ± 0.05 mg/g), flavonoid (62.82 ± 0.07 mg/g), and ascorbic acid (52.46 ± 0.1 mg/g) components were present in high amounts, while β-carotene (62.92 ± 0.02 µg/mg) and lycopene (60.42 ± 0.8 µg/mg) were present in lower amounts. The antimicrobial proficiency of modified solvent-assisted extract was evaluated against four pathogenic bacterial and one fungal strain, namely *Staphylococcus* *aureus* (MTCC 3160), *Klebsiella* *pneumoniae* (MTCC 3384), *Pseudomonas* *aeruginosa* (MTCC 2295), and *Salmonella* *typhimurium* (MTCC 1254), and *Candida* *albicans* (MTCC 183), respectively. The zone of inhibition was comparable to antibiotics streptomycin and amphotericin were used as a positive control for pathogenic bacterial and fungal strains. The extract showed significantly higher (*p* < 0.05) anti-inflammatory activity during the albumin denaturation assay (43.56–86.59%) and HRBC membrane stabilization assay (43.62–87.69%). The extract showed significantly (*p* < 0.05) higher DPPH (2,2-diphenyl-1-picrylhydrazyl) scavenging assay and the obtained results are comparable with BHA (butylated hydroxyanisole) and BHT (butylated hydroxytoluene) with percentage inhibitions of 82.46%, 83.34%, and 84.23%, respectively. Therefore, the obtained results concluded that ethanolic extract of *Woodfordia fruticosa* flowers could be utilized as a magnificent source of phenols used for the manufacturing of value-added food products.

## 1. Introduction

Inflammation is an insusceptible framework reaction of the immune system against harmful stimuli like pathogenic microbes, harmed cells, toxic compounds, or irradiation and acts by eliminating destructive stimuli and recuperating the system [1]. Inflammation, therefore, is a resistance mechanism that is essential to health [2]. However, when it becomes excessive and persists for a long time, it results in numerous pathological conditions such as septic shock and rheumatoid arthritis [3]. Therefore, to manage hyper inflammation conditions and get rid of inflammatory disease, several steroidal and non-steroidal anti-inflammatory drugs and immunosuppressants are recommended by the physician. Due to these synthetic drugs, a person requires long-term treatment, and these drugs are primarily associated with adverse side effects due to the inhibition of cyclooxygenase isozymes COX-1 and COX-2, which catalyze the transformation of arachidonic acid into prostaglandins and thromboxane [4]. The prostaglandins and thromboxane play an important role in vasodilation and platelet adhesion. The inhibition of cyclooxygenase isozymes brings about the resistance of their synthesis and accordingly the drugs antagonistically influence the gastric mucosa, renal, cardiovascular, hepatic, and hematologic system [5]. Besides, the resistance of infection-causing microorganisms against antibiotics has emerged as a serious threat to humankind. These synthetic antibiotics, especially in developing nations, are expensive and deficient for the treatment of diseases caused by pathogenic microorganisms and have incidental effects and adulterations like edema, heaving, queasiness, diarrhea, coughing, abdominal pain, and loss of appetite [6]. Therefore, to reduce the severe adverse effects of synthetic drugs used for the treatment of inflammation and against pathogenic microorganisms and to fill the therapeutic gap as well as to compensate for the reaction required during the secondary treatment, scientists and researchers are more focused on naturally occurring products nowadays. In the northern Himalayan region, there are several unexplored species of plants that are used by the local people traditionally for health care. Ethnopharmacological *Woodfordia fruticose* is belongs to the family Lythraceae and is locally known as Dhatki, Dawi, Dhai, Dhavdi, etc., and it is used to cure various disorders that include immunomodulatory, antitumor, hepatoprotective, and antiulcer activity, as well as urinary disorders, wounds, diarrhea, bleeding injuries, and headache [7]. Traditionally, dried flowers have been used to treat the disorders of mucus membrane and paste prepared from flowers is used for the treatment of asthma and cough [8]. According to the Ayurveda study of medicine, the flowers exhibit the properties to suppress Kapha and Pitta [9]. Due to wound healing, analgesic, antirheumatic, and anthelmintic properties, the flowers are extensively used for medicinal purposes by tribal people [10]. The dried flower of *Woodfordia fruticosa* consists of numerous bioactive compounds such as oenothein B, isochimacoalin-A, and oligomers including woodfordins A, B, C, E, G, H, and I, as well as quercetin [11]. Besides, different examinations using distinctive extraction procedures for isolation of bioactive compounds obligated for biological activities have been performed. These methods, however, are not very compelling and result in the auto-oxidation of organic mixtures [12,13]. In the present study, a modified solvent evaporation technique was utilized to overcome this issue as the procedure incorporates dissemination of organic solvents that gather high energy upon exchange with aligned molecules to escape from bonds to another molecule. This outcome in the detachment of these molecules from the mass of liquid in vapor form with air [14]. In our previous study, a modified solvent evaporation technique was used to assess the bioactivity of mushroom extract that revealed promising results as compared to the vacuum oven drying process. Therefore, the present study was carried out to evaluate the anti-inflammatory, antimicrobial, and antioxidant potential of ethanol extract of *Woodfordia fruticosa* flowers. In the present study, the HRBC (human red blood cell) membrane and albumin denaturation assays were used for evaluation of the anti-inflammatory potential of the extract, as the HRBC membrane is analogous to the lysosomal membrane and stabilization of the membrane indicates that the extract can stabilize the lysosomal membranes [12,15]. Protein denaturation results in the production of autoantigens in certain diseases; thus, to evaluate the anti-inflammatory properties of ethanol extract of *Woodfordia fruticosa* flowers, albumin denaturation assay was performed. Until now, no published report has been available on the extraction of bioactive compounds from *Woodfordia fruticosa* flowers utilizing the modified evaporated solvent technique and evaluation of bioactivity of the extract. Therefore, the present study was carried out with the following objectives: Extraction of ethanolic extract using the modified solvent technique and its characterization using gas chromatography–mass spectroscopy (GC-MS), bioactivity evaluation of the extract in comparison to artificial counterparts, and in vitro antimicrobial and in vitro anti-inflammatory activity of the extract.

## 2. Results and Discussion

### 2.1. GC-MS Analysis

**Figure 1:**
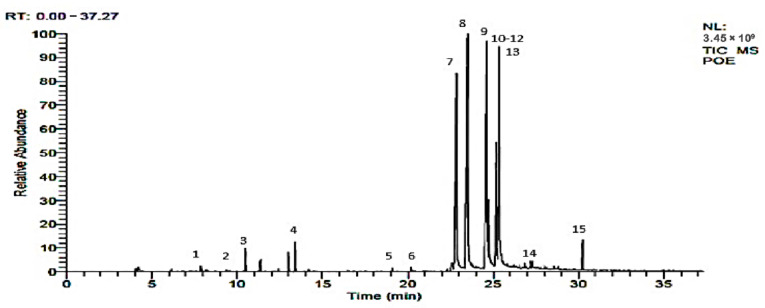
GC-MS chromatogram of *Woodfordia fruticosa* flower extract showing vital bioactive components.

**Figure 2:**
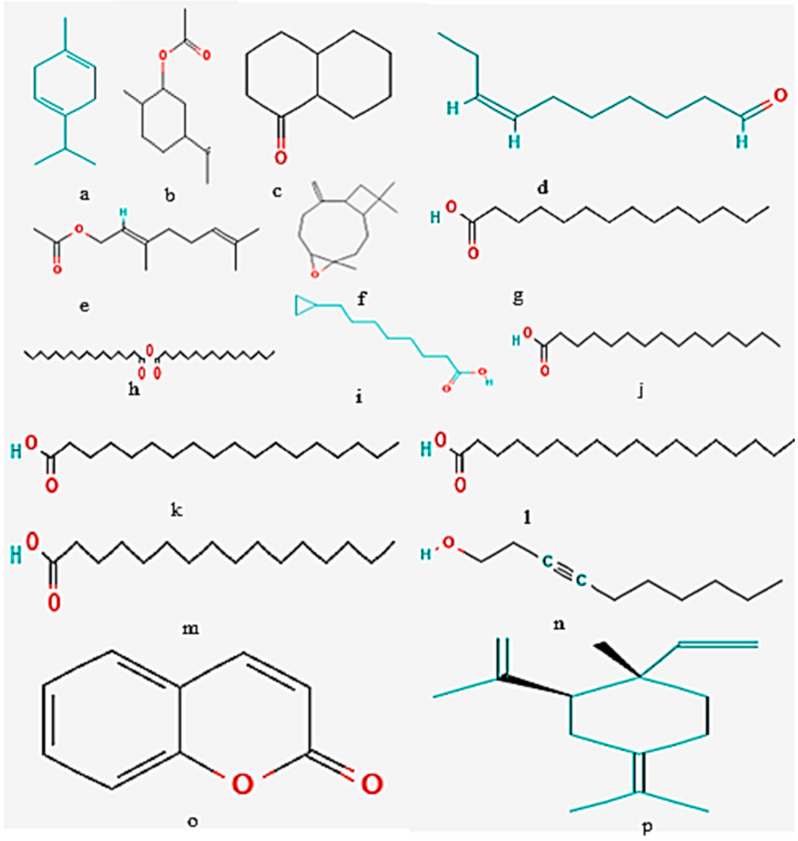
Chemical structure of bioactive components isolated from *Woodfordia fruticosa* flower extract (**a**) **γ**–Terpinene, (**b**) Dihydrocarvyl acetate, (**c**) 1-Decalone (cis-trans), (**d**) cis-7-Decen-1-al, (**e**) 2,6-Octadien-1-ol, 3,7-dimethyl-acetate, (*E*)-(Geranyl acetate), (**f**) Caryophyllene Epoxide, (**g**) Tetradecanoic acid, (**h**) Palmitic anhydride, (**i**) Cyclopropaneoctanoic acid, (**j**) Pentadecanoic acid, (**k**) Pentadecanoic acid, (**l**) Octadecanoic acid, (**m**) n- Hexadecanoic acid (**n**) 3-Decyn-1-ol, (**o**) 2H-1-Benzopyran-2-one, and (**p**) gamma-elemene.

The screening of bioactive compounds from the modified solvent evaporation-assisted ethanolic extract of *Woodfordia fruticosa* flowers revealed the presence of polyphenolic components. The analysis of GC-MS results led to the identification of different types of bioactive compounds from the extract and the results are presented in Figure 1 and Figure 2. The structure of the obtained compound was analyzed based on the fragmentation pattern of mass spectra and their direct comparison with already published mass spectra as well as with the chemical profiles available in NIST (National Institute of Standards and Technology) library, Gaithersburg, Md, D, USA. The chemical profile along with the retention time, peak percentage area, molecular formula, molecular weight, and structure of identified compounds are presented in Table 1. The compounds with a high percentage peak area, namely **γ** -Terpinene, Dihydrocarvyl acetate, 1-Decalone (cis-trans), cis-7-Decen-1-al, Tetradecanoic acid, Palmitic anhydride, Pentadecanoic acid, Octadecanoic acid, n-Hexadecanoic acid, and 3-Decyn-1-ol, 2,6-Octadien-1-ol, 3,7-dimethyl-, acetate, €-(Geranyl acetate), as well as Caryophyllene Epoxide, Cyclopropaneoctanoic acid, Cyclopropaneoctanoic acid, 2H-1-Benzopyran-2-one, 2H-1-Benzopyran-2-one, and gamma-elemene are present in the ethanolic extract of *Woodfordia fruticosa* flowers. Therefore, characterization of ethanol extract by GCMS analysis revealed the presence of secondary metabolic compounds that have therapeutic properties including antimicrobial, antioxidant, anti-inflammatory, and anticancer properties [16].

### 2.2. Antimicrobial Activity

**Figure 3:**
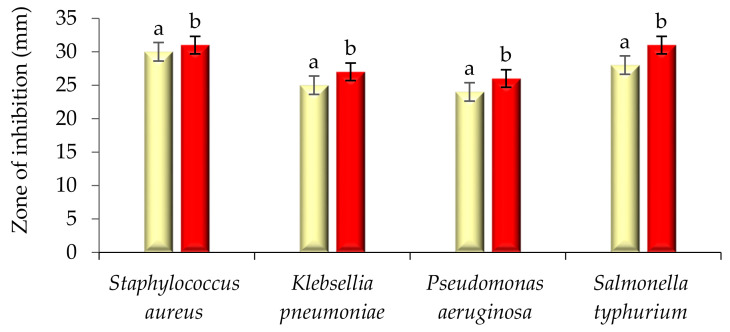
Antibacterial activity (zone of inhibition) of *Woodfordia fruticosa* flower’s dry extract and antibiotic streptomycin against Gram-positive and Gram-negative bacteria. Data are presented as means ± SEM (*n* = 3); a, b: Means within the column with different lowercase superscripts are significantly different (*p* < 0.05).

**Figure 4:**
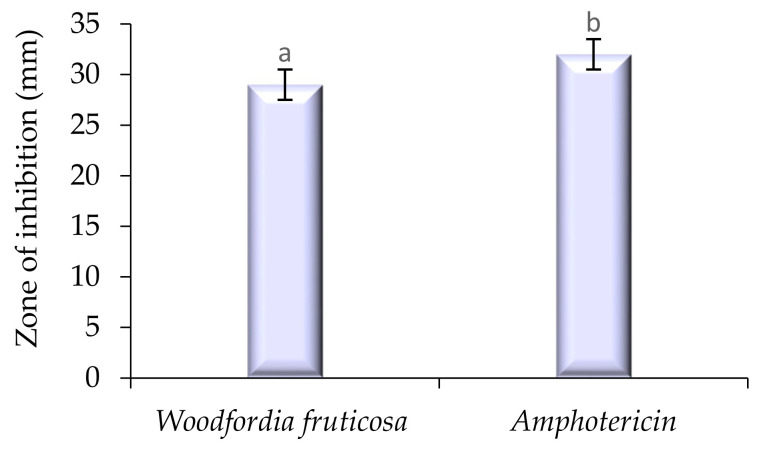
Antifungal activity (zone of inhibition) of *Woodfordia fruticosa* flower’s dry extract against *Candida albicans.* Data are presented as means ± SEM (*n* = 3); a, b: Means within the column with different lowercase superscripts are significantly different (*p* < 0.05).

The modified solvent evaporated *Woodfordia fruticosa* flowers extract was evaluated for its antimicrobial activity against pathogenic bacteria and fungi, and the results are presented in Figure 3 and Figure 4. In the present study, it was observed that the extract showed a significantly higher zone of inhibition against *S. aureus* followed by *Salmonella typhurium, Klebsellia pneumonae,* and *Pseudomonas aeruginosa* with the inhibition zone ranging from 24 ± 0.7 to 30 ± 0.05 mm, respectively. The extract is more active to *S. aureus,* which is because the microorganism is Gram-positive and has a thick hydrophobic structure cell wall. The hydrophobic structure of the cell wall allows for an excellent number of proteins lipids, peptidoglycans, and other biological components present in the extract to pass through the cell membrane [12]. The outer membrane of Gram-negative bacteria consists of a layer of lipopolysaccharides that results in variation in the hydrophobic properties or mutation in porins and other factors, and thus results in the resistance of these bacteria against the extract [17]. Against the pathogenic fungi, *Candida albicans,* the extract showed significantly (*p* < 0.05) good antifungal activity with a zone inhibition diameter of 30.1 ± 0.7 mm. The antifungal activity of ethanol extract is due to the phytochemicals limonene and **γ** -Terpinene. The compounds can diffuse into the cell membrane and resulting in the disruption of cellular components [14]. Results are well in accordance with the findings of Kaur and Kaur [18], who revealed the antimicrobial activity of ethanolic extract of *Woodfordia fruticosa* flowers, respectively.

### 2.3. Time–Kill Study

**Figure 5:**
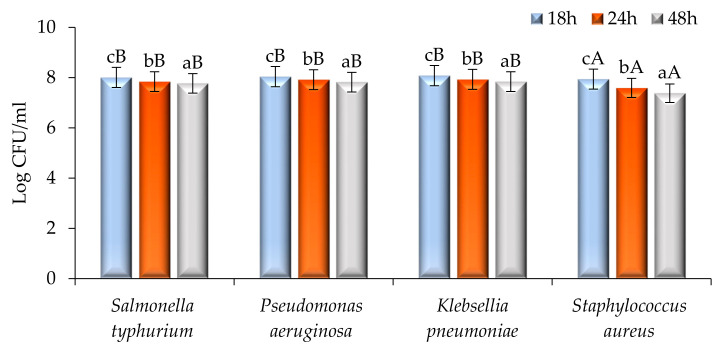
Time–kill kinetics of *Woodfordia fruticosa* flower’s dry extract against the growth of food pathogenic bacteria during different time intervals. Data are presented as means ± SEM (*n* = 3); a–c: Means within the column with different lowercase superscripts are significantly different (*p* < 0.05); A, B: Means within the row with different uppercase superscripts are significantly different (*p* < 0.05) from each other.

**Figure 6:**
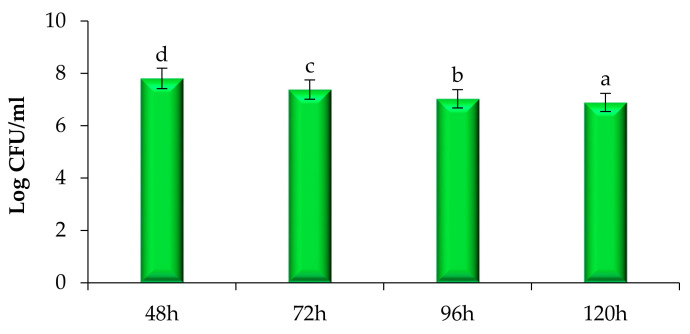
Time–kill kinetics of *Woodfordia fruticosa* flower’s dry extract against the growth of pathogenic *Candida albicans* during different time intervals. Data are presented as means ± SEM (*n* = 3); a–d: Means within the column with different lowercase superscripts are significantly different (*p* < 0.05).

The time–kill study of modified solvent evaporated ethanolic extract of *Woodfordia fruticosa* flowers was performed and results are presented in Figure 5 and Figure 6. Against pathogenic bacteria and fungi, the ethanol extract showed a significantly (*p* < 0.05) higher killing effect with the increase in the time interval. Among all pathogenic bacteria used for the study, *S. aureus* showed a significantly (*p* < 0.05) higher log CFU/mL value reduction (7.94–7.38) followed by *Salmonella typhurium* (8.01–7.77), *P. aeruginosa* (8.04–7.82), and *K.* *pneumoniae* (8.08–7.84). The inhibitory effect against *S. aureus* was due to the presence of secondary metabolites. These secondary metabolites inhibit the biochemical pathway, protein synthesis, and cause disintegration of the outer membrane [17]. *P. aeruginosa* showed significantly higher Log CFU as the outer membrane constitutes an asymmetric bilayer of phospholipids and lipopolysaccharides embedded with channels of β-barrel and porins proteins that prevent the penetration of antibiotics and extract [19]. Another major factor of resistance was bacterial efflux pumps that expel the compounds toxic to the cell, the production of enzymes that inactive the antibiotic or compounds present in the extract by breaking down or modifying their structures, and by the acquisition of resistant genes or by mutational changes [20]. In the case of the pathogenic fungi *Candida albicans,* there was a significant (*p* < 0.05) reduction in the Log CFU/mL value (7.81–6.89). The reduction may be due to the reason that yeast was hydrophilic and also lacks surface-active hydrophobin proteins; therefore, hydrophobic antifungal compounds easily get accumulated at the conidia of the fungal strain [21].

### 2.4. Estimation of Phytochemicals and Their Antioxidant Activity

**Figure 7:**
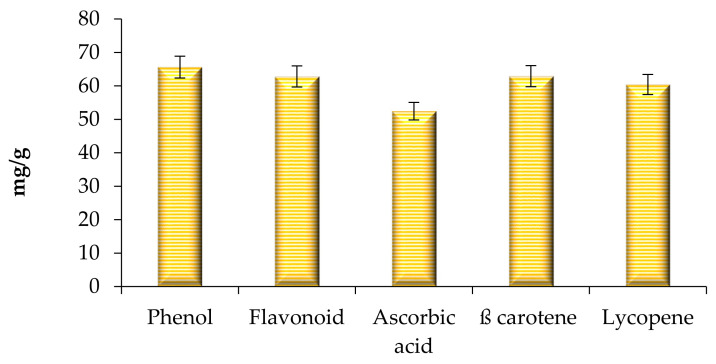
Total phenol, flavonoid, ascorbic acid content, total β-carotene, and lycopene content of modified solvent evaporated *Woodfordia fruticosa* flower extract. Data are presented as means ± SEM (*n* = 3).

**Figure 8:**
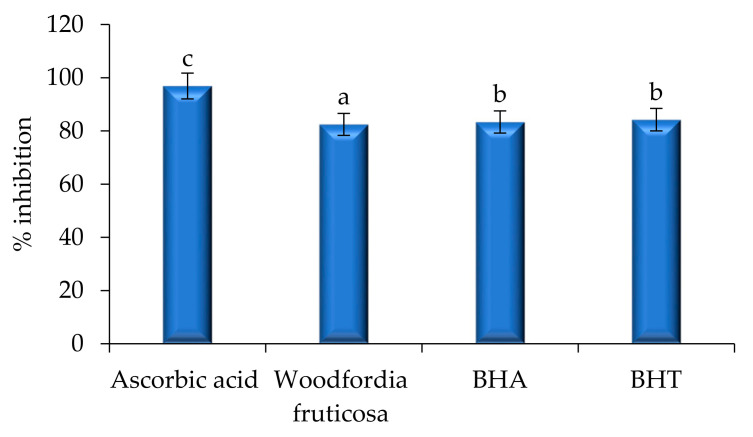
Antioxidant activity of *Woodfordia fruticosa* flower’s dry extract in comparison with natural and synthetic commercially available antioxidant components. Data are presented as means ± SEM (*n* = 3); a–c: Means within the column with different lowercase superscripts are significantly different (*p* < 0.05).

The estimation of bioactive compounds including phenols, flavonoids, ascorbic acid, lycopene, and carotene was carried out and the results are presented in Figure 7. The extract showed a significant amount (*p* < 0.05) of phenol (65.62 ± 0.05 mg/g), flavonoid (62.82 ± 0.07 mg/g), ascorbic acid (52.46 ± 0.1 mg/g), β-carotene (62.92 ± 0.02 mg/g), and lycopene (60.42 ± 0.8 mg/g), respectively. The antioxidant activity of the extract was evaluated and the results are shown in Figure 8. Herein, the extract, BHA, BHT, and L-ascorbic acid showed significantly (*p* < 0.05) higher percentage inhibitions, with a percentage inhibition of 82.46%, 83.34%, 84.23%, and 96.89% respectively. The antioxidant activity of the extract was due to the presence of phenolic acid, flavonol glycosides, gallates, and tannins [12,14]. In this context, Ghante et al. [8], Banerjee and De [22]; and Shubha and Bhatt [10] reported an excellent number of phenolic compounds and DPPH scavenging activity of *Woodfordia fruticosa* flowers to extract. 

### 2.5. Anti-Inflammatory Properties

**Figure 9:**
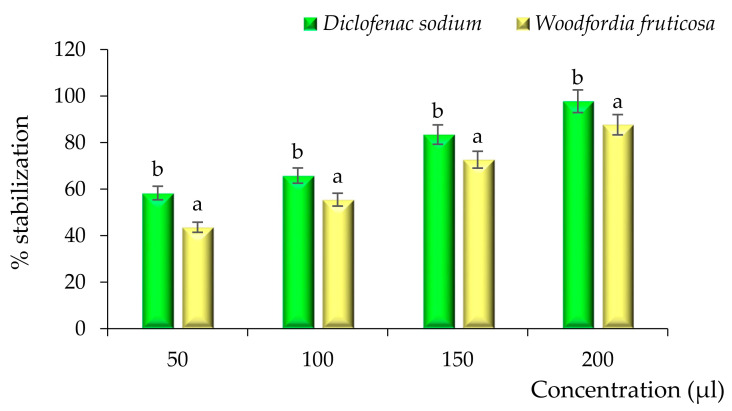
Anti-inflammatory activity of *Woodfordia fruticosa* flower dry extract in comparison with standard diclofenac sodium during the HRBC membrane stabilization assay. Data are presented as means ± SEM (*n* = 3); a, b: Means within the column with different lowercase superscripts are significantly different (*p* < 0.05).

**Figure 10:**
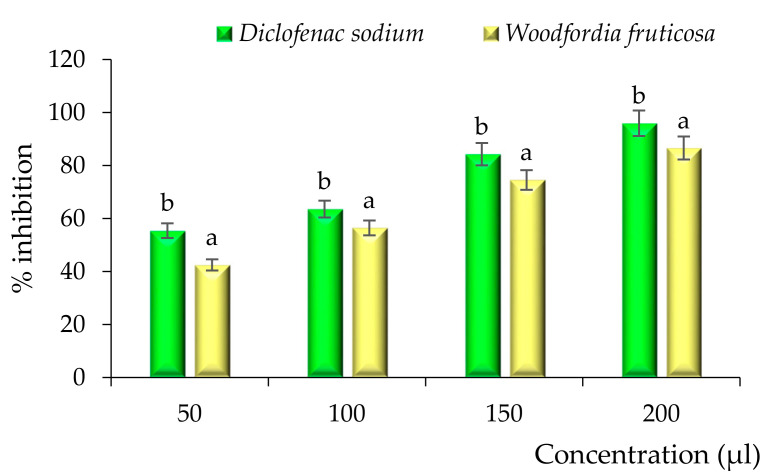
Anti-inflammatory activity of *Woodfordia fruticosa* flower dry extract in comparison with standard diclofenac sodium during the albumin denaturation assay. Data are presented as means ± SEM (*n* = 3); a, b: Means within the column with different lowercase superscripts are significantly different (*p* < 0.05).

Evaluation of the anti-inflammatory activity of the solvent evaporated ethanol extract of *Woodfordia fruticosa* flowers was done by HRBC membrane stabilization and albumin denaturation assay, and diclofenac sodium was taken as a positive control. The results are represented in Figure 9 and Figure 10. During both assays, a significant (*p* < 0.05) percentage stabilization and percentage inhibition were observed between the standard and ethanol extract of *Woodfordia fruticosa* flowers. Standard diclofenac sodium showed stabilization ranges from 58.32 to 97.78% and 43.62 to 87.69% for the HRBC membrane stabilization assay. Likewise, standard diclofenac sodium showed a percentage inhibition ranging from 55.42 to 95.93% and 43.56 to 86.59% for the albumin denaturation assay. In the present study, the HRBC membrane stabilization assay is considered for limiting inflammation response as the membrane is analogous to the lysosomal membrane [23]. In addition to this, the denaturation of protein is well correlated with the inflammatory response and results in various inflammatory diseases. Injuries of tissues may refer to the denaturation of protein constituted by cells or by intracellular substances [24,25]. Ethanol extract of *Woodfordia fruticosa* flowers was supposed to inhibit the synthesis or release of major inflammatory mediators and stabilization of cell membranes [7]. The anti-inflammatory effects can be correlated with the essential bioactive compounds isolated from the extract, namely Octadecanoic acid, n-Hexadecanoic acid, and **γ** -Terpinene. These bioactive compounds have been reported to possess anti-inflammatory activity.

## 3. Materials and Methods

Fresh *Woodfordia fruticosa* flowers were collected in August 2020 from the forest of Mandi, Himachal Pradesh, India, and submitted to the herbarium Department of Botany, Punjab University, Chandigarh (Voucher specimen number 35329). Chemicals of analytic grade including ethanol, phosphoric acid, Muller Hinton Agar (MHA), Sabouraud Dextrose Agar (SDA), and Nutrient Agar were procured from Hi-Media, Private Limited, Mumbai, India. This research was primarily focused on antibacterial and antifungal activity of the extract; therefore, standard pathogenic strains of both Gram-positive and Gram-Negative bacteria including *Staphylococcus aureus* (MTCC 3160) *Klebsiella pneumoniae* (MTCC 3384), *Pseudomonas aeruginosa* (MTCC 2295), and *Salmonella typhmurium* (MTCC 1254), as well as one fungal strain of *Candida albicans* (MTCC 183), were obtained from Microbial Type Culture Collection (MTCC) IMTECH, Chandigarh, India. Chromatography grade chemicals including ethanol and methanol were purchased from Thermo Fisher Scientific Limited, Mumbai, India. DMSO (Dimethyl sulfoxide ≥ 99.9%), Folin–Ciocalteu’s reagent (2N), DPPH (2,2-diphenyl-1-picrylhydrazyl), and TFA (trifluoroacetic acid 99%) were purchased from Sigma-Aldrich (St. Louis, MO, USA). Aluminum chloride 6-hydrate, ammonium molybdate tetrahydrate, sodium carbonate anhydrous, tri-sodium phosphate 12-hydrate, BHA (butylated hydroxyanisole), BHT (butylated hydroxytoluene), sodium hydroxide pellets, and sodium nitrite were procured from Hi-Media Private Limited, Mumbai, India.

### 3.1. Preparation of Flower Extract

Freshly collected flowers were washed with double distilled water to remove dust and other pollutants and flowers were then kept at 30 °C in a hot air oven (SGMlab Solutions Private Limited, India) to remove the water content. The dried flowers were ground to a fine powder using a mechanical grinder (Phillips, mixer grinder, 900 watts, New Delhi, India) and powder was then used for the preparation of extract. Briefly, a 40 g sample was dispersed in 400 mL of absolute ethanol (1:10 *w*/*v* ratio) in a conical flask, and the flask containing the mixture was kept in an orbital shaker (Thermo Fisher Scientific Pvt. Ltd., Mumbai, India) for 72 h. The sample was then filtered using Whatman Filter Paper No. 1 and then kept in a refrigerator (4–7 °C) for the evaporation of ethanol for 72 h. Herein, the flower extract was dispersed in ethanol at refrigerated temperature and molecules of ethanol exchanged the kinetic energy with neighbor molecules to escape from bonds with each other, and every ethanol molecule collected enough energy to leave the mass of liquids and join the air as a vapor. The dried extract obtained was stored at −20 °C in an airtight amber-colored glass vial for further analysis [24].

### 3.2. GC-MS Analysis of the Extract

The separation and identification of essential oil components were achieved using gas chromatography–mass spectrometry (Thermo Fisher Scientific imported from Waltham, MA, USA) coupled with TriPlus RSH autosampler, GC 1300 gas chromatography, and TSQ Duo Mass Selective quadrupole detector. The equipped column was TG-5MS had 40 m length, 0.15 mm internal diameter, and 0.15 µm film thickness. The derivatized samples were diluted in n-hexane with 1:99 ratios and subjected to instrument for analysis in splitless mode. The injector temperature was kept at 250 °C, and the GC program was started from 60 °C for 1 min to 180 °C for 3 min, and the final temperature was achieved to 240 °C for 12 min. The ramp rate was kept constant at 10 °C per min for a complete analysis of 34 min. The program was run at a constant flow of 0.7 mL/min of helium gas. The ion source temperature was operated at 230 °C along with 250 °C of transfer line temperature, and ionization and fragmentation of separated components were accomplished by electron impact at 70 eV. The mass filter was set to scan between *m/z* 45 and 450. The chromatographic and mass spectra data were processed using Xcalliber Software (Thermo Fisher Scientific imported from Waltham, MA, USA).

### 3.3. In Vitro Antimicrobial Assay

In vitro antimicrobial activity of the extract was assessed against pathogenic Gram-positive and Gram-negative bacteria and fungi including *Staphylococcus aureus*, *Klebsiella pneumonia*, *Pseudomonas aeruginosa*, *Salmonella typhimurium*, and *Candida albicans*, respectively. The antimicrobial assay was performed using the agar well diffusion method proposed by Bains and Chawla [12]. Herein, Muller Hinton Agar enriched with 4% NaCl was used to inoculate bacterial strain (1.5 × 10^8^ cells/mL), and Sabouraud Dextrose agar plate was used to inoculate the fungal strain. The wells were made on plates using cork borer wells. The flower extract (10 mg) was dissolved in 10 mL of DMSO (5%) and poured (50 µL) into an agar well with the help of a micropipette. The commercially available antibiotic, streptomycin (1 µg/mL), was used as a positive control, and DMSO was used as a negative control. The MHA plates were inoculated with pathogenic bacterial strains and incubated at 37 °C for 24 h, and the SDA agar plate inoculated with the fungal strain was incubated at 27 °C for 72 h. Results of antimicrobial activity were measured as the zone of inhibition in mm.

### 3.4. Time–Kill Study

A time–kill study was performed by the method followed by Chawla et al. [26]. Herein, 100 µL of flower extract solution was used for all pathogenic microbial strains. For the bacterial strains, the sample was examined after an interval of 0, 18, 24, and 48 h; however, for the fungal strain, the examination was done after a time interval of 48–120 h respectively. The samples of both bacterial and fungal strains were spread on plates containing MHA and SDA and incubated for 37 °C for 24 h and 27 °C for 72 h, respectively. Results were obtained after calculating Log CFU/mL for each sample.

### 3.5. Quantification of Bioactive Compounds 

#### 3.5.1. Total Phenolic Content

Total phenolic content was evaluated by the method proposed by Najda et al., [27]. Briefly, the stock solution of extract (10 mg/10 mL) was prepared in ethanol, and 0.2ml of flower extract was mixed in 1N Folin–Ciocalteu reagent (1 mL) and 2 mL (7.5%) sodium carbonate solution. The reaction mixture was kept undisturbed for 30 min under dark conditions. After 30 min of incubation under dark conditions, absorbance was measured at 760 nm under a UV-visible spectrophotometer (Pharma Test Apparatebau AG, Hainburg, Germany). Gallic acid equivalent (GAE) was used as standard and total phenolic content was expressed in GAE/g dried extract.

#### 3.5.2. Total Flavonoid Content

The evaluation of total flavonoid content was carried out by the proposed method of Bains and Chawla [12]. Briefly, 2 mL of extract was mixed in 5% sodium nitrite (200 µL) and then kept undisturbed for 5 min. Aluminum chloride (10 %, 200 µL) was then added and mixed evenly using a vortex shaker. The reaction mixture obtained was kept undisturbed for 6 min, and 1 M NaOH (2 mL) was added to it. The absorption was determined immediately at 510 nm using a double beam UV-Visible spectrophotometer. Quercetin was used as standard and used to plot calibration curves at different concentrations. The results were expressed as milligrams of quercetin equivalent (QEs)/g of extract.

#### 3.5.3. The Total Ascorbic Acid Content

The ascorbic acid evaluation present in the extract was calculated by the method proposed by Najda et al., [27]. Herein, 100 mg extract of flowers were mixed in 1% metaphosphoric acid at 30 °C constant for 45 min. After 45 min, the mixture was filtered using Whatman no. 1 filter paper and the filtrate was obtained dissolved in 2,6 dichlorophenol. The absorbance of the reaction mixture was then observed at 515 nm within 30 min. L-ascorbic acid was used as the standard to calculate the total amount of ascorbic acid.

#### 3.5.4. β-Carotene and Lycopene Determination

Dried ethanol extract (100 mg) was dissolved in 10 mL of the acetone–hexane mixture (4:6) and kept undisturbed at 27 °C for 1 min. The obtained mixture was then filtered using Whatman no. 1 filter paper, and absorbance was measured at three different wavelengths such as 453, 505, and 663 nm, respectively [13]. The total β-carotene and Lycopene values were calculated by applying the following equations:

(1) $$\beta -\mathrm{carotene}\;\left(\mathrm{mg}/100\;\mathrm{mg}\right)\;=\;0.216{\mathrm{Abs}}_{663}\;-\;0.304{\mathrm{Abs}}_{505}\;+\;0.452{\mathrm{Abs}}_{453}\;\left(X\right)$$

(2) $$\mathrm{Lycopene}\;\left(\mathrm{mg}/100\;\mathrm{mg}\right)\;=\;-0.0458{\mathrm{Abs}}_{663}\;+\;0.372{\mathrm{Abs}}_{505}\;-\;0.0806{\mathrm{Abs}}_{453}\;\left(Y\right)$$

### 3.6. Antioxidant Activity

#### DPPH Free Radical Scavenging Assay

The method proposed by Sadh et al., [28] was used to evaluate antioxidant activity using DPPH assay. Briefly, the stock solution of extract (10 mg/10 mL) was prepared in ethanol, and 0.2 mL of flower extract was dissolved in 0.1 mM DPPH solution (2 mL) in a test tube and kept constant for 30 min in dark conditions. A change in the color of DPPH from purple to pale yellow was observed, and the absorbance of the solution was then measured at 517 nm. Herein, the extract was compared with standard natural and synthetic antioxidants for antioxidant activity. The percentage of free radical scavenging activity was calculated using the following equation:

(3) $$\mathrm{Inhibition}\;\left(\%\right)\;=\left(\frac{\mathrm{Absorbance}\;\mathrm{of}\;\mathrm{DPPH}\;-\;\mathrm{Absorbance}\;\mathrm{of}\;\mathrm{sample}}{\mathrm{Absorbance}\;\mathrm{of}\;\mathrm{DPPH}}\right)\times 100$$

### 3.7. Anti-Inflammatory Properties

#### 3.7.1. HRBC Membrane Stabilization Assay

Blood (5 mL) from healthy volunteers not taking nonsteroidal anti-inflammatory drugs (NSAID) for 15 days was used to perform HRBC membrane stabilization. An equal volume of Alsever solution (20.5 g dextrose, 8 g sodium citrate, 0.55 g citric acid, and 4.2 g sodium chloride in 1000 mL water) and blood was dissolved and centrifuge at 3000× *g* for 15 min. After centrifugation, the packed cell obtained was washed with isosaline solution. The assay solution containing 500 µL (mg/mL concentration) flower extract, 500 µL HRBC suspension, 0.15 M 1 mL phosphate buffer with pH 7.4, and 2 mL of 0.36% hypo saline solution was prepared. The mixture was incubated for 30 min at 37 °C in the BOD (biological oxygen demand) incubator, and the suspension was centrifuged at 3000× *g* for 20 min. Deionized water and diclofenac sodium were used as a negative and positive control, respectively [14].

The percentage HRBC membrane stabilization was calculated as follows:

(4) $$\mathrm{Inhibition}\;(\%)\;=\;100\;-\;\left(\frac{\mathrm{Oq}}{\mathrm{Or}}\times 100\right)$$

where Oq is the optical density of the sample and Or is the absorbance of the control.

#### 3.7.2. Albumin Denaturation Assay

Albumin denaturation assay was performed by method followed by Bains and Chawla [12]. Herein, 5 mL volume of the reaction mixture containing 200 µL of fresh egg albumin, phosphate buffer solution (2.8 mL) with pH 6.4, and 2 mL of flower extract was prepared. The mixture was incubated in a BOD incubator for 15 min at 37 °C followed by heating up to 70 °C for 5 min, and the absorbance was measured at 660 nm. Diclofenac sodium and deionized water were used as a positive and negative control, respectively. Percentage inhibition by albumin denaturation assay was calculated as follow:

(5) $$\mathrm{Inhibition}\;(\%)\;=\;100\;\times \;\left(\mathrm{Ay}/\mathrm{Az}-1\right)$$

where Ay is the absorbance of the test sample and Az is the absorbance of the control.

### 3.8. Statistical Analysis

Statistical analysis of observed results was done by following the method proposed by Kaushik et al., [29]. The standard error mean was calculated by using Microsoft Excel Office, 2016 (Microsoft Corporation, WA, USA), and the statistical difference was calculated using one-way ANOVA (analysis of variance), and comparison between the means was calculated by the difference value.

## 4. Conclusions

In the present study, the solvent evaporated ethanol extract of *Woodfordia fruticosa* flowers was taken to evaluate the anti-inflammatory, antimicrobial, and antioxidant activity. The extract showed a significantly (*p* < 0.05) higher zone of inhibition against Gram-positive bacteria *S. aureus*, followed by *K. pneumonia*, *P. aeruginosa,* and *E. coli,* respectively. Ethanol extract, BHT, and BHA showed non-significant differences (*p* < 0.05) during the DPPH free radical scavenging assay. The extract showed a significant amount of phenolic, flavonoid, ascorbic acid, β-carotene, and lycopene content, respectively. The extract also showed potential anti-inflammatory properties during human red blood cell membrane stabilization and albumin denaturation assay. The HRBC membrane is analogous to the lysosomal membrane, which inhibits the release of lysosomal constitutes of activated neutrophils that are responsible for inflammation. Furthermore, *Woodfordia fruticosa* flower extract, BHT, and BHA showed a non-significant (*p* > 0.05) difference in terms of percentage inhibition during the DPPH scavenging assay. The saturated fatty acids, hexadecanoic acids, and other secondary metabolites identified during GC-MS analysis are attributed to a wide range of biological activities including anticancer, antimicrobial, antioxidant, and anti-inflammatory activity. Therefore, it is concluded from the present study that *Woodfordia fruticosa* is an unexplored wild-growing plant that constitutes numerous bioactive compounds responsible for different biological activity and, hence, can be utilized for the preparation of nutraceutical products and in food industries.

## Figures and Tables

**Table 1:** Phyto-compounds identified in modified evaporated ethanol solvent extract of *Woodfordia fruticosa* flower.

Peak Number	Retention Time (Min)	Area %	Molecular Weight (g)	Molecular Formula	Compound
1	7.86	0.36	136	C_10_H_16_	**γ**-Terpinene
2	8.20	0.10	196	C_12_H_20_O_2_	Dihydrocarvyl acetate
3	10.47	1.26	152	C_10_H_16_O	1-Decalone (cis-trans)
4	12.43	0.13	154	C_10_H_18_O	*cis*-7-Decen-1-al
5	13.39	1.35	196	C_12_H_20_O_2_	2,6-Octadien-1-ol, 3,7-dimethyl-, acetate, (*E*)-(Geranyl acetate)
6	16.51	0.30	220	C_15_H_24_O	Caryophyllene Epoxide
7	20.19	0.31	228	C_14_H_28_O_2_	Tetradecanoic acid
8	22.85	19.12	494	C_32_H_62_O_3_	Palmitic anhydride
9	23.50	22.67	282	C_11_H_20_O_2_	Cyclopropaneoctanoic acid
10	24.75	2.42	242	C_15_H_30_O_2_	Pentadecanoic acid
11	24.75	2.42	284	C_18_H_36_O_2_	Octadecanoic acid
12	24.75	2.42	256	C_16_H_32_O_2_	*n*-Hexadecanoic acid
13	25.16	7.46	154	C_10_H_18_O	3-Decyn-1-ol
14	27.19	0.25	260	C_9_H_14_O_2_	2*H*-1-Benzopyran-2-one
15	30.23	1.64	204	C_15_H_24_	gamma-Elemene

## Data Availability

Data sharing is not applicable to this article.

## Sample Availability

Fresh *Woodfordia fruticosa* flowers were collected in August 2020 from the forest of Mandi, Himachal Pradesh, India, and submitted to the herbarium Department of Botany, Punjab University, Chandigarh (Voucher specimen number 35329).

## Abbreviations

GC-MS	Gas chromatography–mass spectroscopy
HRBC	Human red blood cell
DPPH	2,2-diphenyl-1-picrylhydrazyl
BHA	Butylated hydroxyanisole
BHT	Butylated hydroxytoluene
MTCC	Microbial Type Culture Collection

## References

[1] Chen L., Deng H., Cui H., Fang J., Zuo Z., Deng J., Li Y., Wang X., Zhao L. (2018). Inflammatory responses and inflammation-associated diseases in organs. Oncotarget.

[2] Tottoli E.M., Dorati R., Genta I., Chiesa E., Pisani S., Conti B. (2020). Skin wound healing process and new emerging technologies for skin wound care and regeneration. Pharmaceutics.

[3] Edilova M.I., Akram A., Abdul-Sater A.A. (2021). Innate immunity drives pathogenesis of rheumatoid arthritis. Biomed. J..

[4] Ferrer M.D., Busquets-Cortés C., Capo X., Tejada S., Tur J.A., Pons A., Sureda A. (2019). Cyclooxygenase-2 inhibitors as a therapeutic target in inflammatory diseases. Cur. Med. Chem..

[5] Grosser T., Smyth E., FitzGerald G.A. (2011). Anti-inflammatory, antipyretic, and analgesic agents; pharmacotherapy of gout. Goodman Gilman’s Pharmacol. Basis Ther..

[6] Karthikeyan G., Swamy M.K., Viknesh M.R., Shurya R., Sudhakar N., Swamy M.K. (2020). Bioactive Phytocompounds to Fight Against Antimicrobial Resistance. Plant-Derived Bioactives.

[7] Baravalia Y., Vaghasiya Y., Chanda S. (2012). Brine shrimp cytotoxicity, anti-inflammatory and analgesic properties of *Woodfordia fruticosa* Kurz flowers. Iran. J. Pharm. Res..

[8] Hiralal Ghante M., Bhusari K.P., Duragkar N.J., Ghiware N.B. (2014). Pharmacological evaluation for anti-asthmatic and anti-inflammatory potential of *Woodfordia fruticosa* flower extracts. Pharmaceut. Biol..

[9] Roy A., Janbandhu S. (2020). An ethnobotanical analysis on flora-medicine continuum among the tribal inhabitants of Ratnagiri and Palghar district, Maharashtra, India. Ethnobot. Res. Appl..

[10] Shubha J.R., Bhatt P. (2021). Functional attributes of polyphenol-rich Woodfordia fruticosa extract: An active ingredient in traditional Indian medicine with nutraceutical potential. J. Herb. Med..

[11] Verma N., Amresh G., Sahu P.K., Mishra N., Rao C.V., Singh A.P. (2013). Wound healing potential of flowers extracts of *Woodfordia fruticosa* Kurz. Indian J. Biochem. Biophys..

[12] Bains A., Chawla P. (2020). In vitro bioactivity, antimicrobial and anti-inflammatory efficacy of modified solvent evaporation assisted *Trametes versicolor* extract. Biotech.

[13] Bains A., Chawla P., Tripathi A., Sadh P.K. (2021). A comparative study of antimicrobial and anti-inflammatory efficiency of modified solvent evaporated and vacuum oven dried bioactive components of *Pleurotus floridanus*. J. Food Sci. Technol..

[14] Malik A., Najda A., Bains A., Nurzyńska-Wierdak R., Chawla P. (2021). Characterization of *Citrusnobilis* Peel Methanolic Extract for Antioxidant, Antimicrobial, and Anti-Inflammatory Activity. Molecules.

[15] Bains A., Tripathi A. (2017). Evaluation of antioxidant and anti-inflammatory properties of aqueous extract of wild mushrooms collected from Himachal Pradesh. Asian J. Pharm. Clin. Res..

[16] Dubey D., Patnaik R., Ghosh G., Padhy R.N. (2014). In vitro antibacterial activity, gas chromatography–mass spectrometry analysis of *Woodfordia fruticosa* Kurz. leaf extract and host toxicity testing with in vitro cultured lymphocytes from human umbilical cord blood. Osong. Public Health Res. Perspect..

[17] Kebede T., Gadisa E., Tufa A. (2021). Antimicrobial activities evaluation and phytochemical screening of some selected medicinal plants: A possible alternative in the treatment of multidrug-resistant microbes. PLoS ONE.

[18] Kaur R., Kaur H. (2010). The Antimicrobial activity of essential oil and plant extracts of *Woodfordia fruticosa*. Arch. Appl. Sci. Res..

[19] Kepiro I.E., Marzuoli I., Hammond K., Ba X., Lewis H., Shaw M., Ryadnov M.G. (2019). Engineering chirally blind protein pseudocapsids into antibacterial persisters. ACS Nano.

[20] Venter H. (2019). Reversing resistance to counter antimicrobial resistance in the World Health Organisation’s critical priority of most dangerous pathogens. Biosci. Rep..

[21] Paraszkiewicz K., Moryl M., Płaza G., Bhagat D.K., Satpute S., Bernat P. (2021). Surfactants of microbial origin as antibiofilm agents. Int. J. Environ. Health Res..

[22] Banerjee A., De B. (2014). Antioxidant activity of ethnomedicinally used flowers of West Bengal, India. Int. J. Pharmacogn. Phytochem. Res..

[23] Sharma S., Kota K., Ragavendhra P. (2018). HRBC Membrane Stabilization as a study tool to explore the Anti Inflammatory activity of Alliumcepa Linn.–Relevance for 3R. J. Adv. Med. Dent. Sci. Res..

[24] Osman N.I., Sidik N.J., Awal A., Adam N.A.M., Rezali N.I. (2016). In vitro xanthine oxidase and albumin denaturation inhibition assay of *Barringtonia racemosa* L. and total phenolic content analysis for potential anti-inflammatory use in gouty arthritis. J. Intercult. Ethnopharmacol..

[25] Chawla P., Kumar N., Kaushik R., Dhull S.B. (2019). Synthesis, characterization and cellular mineral absorption of nanoemulsions of *Rhododendron arboreum* flower extracts stabilized with gum arabic. J. Food Sci. Technol..

[26] Chawla P., Najda A., Bains A., Nurzyńska-Wierdak R., Kaushik R., Tosif M.M. (2021). Potential of Gum Arabic Functionalized Iron Hydroxide Nanoparticles Embedded Cellulose Paper for Packaging of Paneer. Nanomaterials.

[27] Najda A., Klimek K., Piekarski W. (2020). Zawartość wybranych metabolitów wtórnych i zdolność przeciwutleniająca ziela *Mentha× piperita* L. var. officinalis Sole f. pallescens Camus suszonego próżniowo. Przemysł Chemiczny..

[28] Sadh P.K., Chawla P., Duhan J.S. (2018). Fermentation approach on phenolic, antioxidants and functional properties of peanut press cake. Food Biosci..

[29] Kaushik R., Chawla P., Kumar N., Janghu S., Lohan A. (2018). Effect of premilling treatments on wheat gluten extraction and noodle quality. Food Sci. Technol. Int..

